# Risk factors for mortality among patients admitted with upper gastrointestinal bleeding at a tertiary hospital: a prospective cohort study

**DOI:** 10.1186/s12876-017-0712-8

**Published:** 2017-12-20

**Authors:** Sibtain M. Moledina, Ewaldo Komba

**Affiliations:** Department of Internal Medicine, Muhimbili University of Health and Allied Sciences, P.O. Box 65001, Dar es Salaam, Tanzania

**Keywords:** Upper gastrointestinal bleeding mortality Tanzania

## Abstract

**Background:**

Upper gastrointestinal bleeding (UGIB) is a common gastrointestinal emergency, which is potentially fatal. Proper management of UGIB requires risk-stratification of patients which can guide the type and aggressiveness of management. The aim of this was study was identify the causes of UGIB and factors that increase the risk of mortality in these patients.

**Methods:**

This was a prospective cohort study conducted over a period of seven months at a tertiary hospital. Adults admitted with UGIB were included in the study. Demographic data, laboratory parameters and endoscopic findings were recorded. Patients were then followed up for 60 days to identify the occurrence of mortality. Chi-square tests and cox-regression was used to determine association between risk factors and mortality in the bivariate and multivariate analysis, respectively.

**Results:**

A total of 170 patients with UGIB were included. Males accounted for the majority (71.2%). Median age of the study population was 40.0 years. Chronic liver disease was present in 30.6% of study patients. The most common cause of UGIB among the 86 patients who underwent endoscopy was oesophageal varices (57%), followed by peptic ulcer disease (18%) and gastritis (10%). Mortality occurred in 57 patients (33.5%) and was significantly higher in patients with high white blood cell count (HR 2.45, p 0.011), raised serum alanine aminotransferase (HR 4.22, p 0.016), raised serum total bilirubin (HR 5.79, p 0.008) and lack of an endoscopic procedure done (HR 4.40, *p* <0.001). Rebleeding was reported in 12 patients (7.1%) and readmission due to UGIB in 4 patients (2.4%)

**Conclusions:**

Oesophageal varices was the most common cause of UGIB. One-third of patients admitted with upper gastrointestinal bleeding died within 60 days of admission, signifying a high burden. Rebleeding and readmission rates were low. A high WBC count, raised serum ALT, raised serum total bilirubin and a lack of endoscopy were independent predictors of mortality. These findings can be used to risk-stratify patients who may benefit from early and more aggressive management.

## Background

Upper Gastrointestinal Bleeding is defined as hemorrhage originating from anywhere between the esophagus and the ligament of Treitz [1]. It is one of the most common gastrointestinal emergencies, with an average mortality rate of 10% [2, 3]. Despite advances in the diagnosis and management of UGIB, the mortality rate has not changed significantly in the last 50 years [2–4].

Effective management requires use of a risk-stratification tool to categorise patients into low-risk and high-risk group, which can be used to guide treatment and follow up [3].

Many risk factors for mortality have been found from different studies. A number of risk scores have been developed also. The most frequently used risk scoring system is the Rockall score which was developed in 1996. This score assesses the risk of death following UGIB and incorporates the patient’s age, systolic blood pressure, heart rate, presence of other comorbidities and endoscopic findings [3]. Another frequently used scoring system is the Glasgow Blatchford Score [3].

Upper GI bleeding continues to be a significant cause of morbidity and mortality in Tanzania. For example, a one restrospective study enrolled 130 admitted patients with UGIB within two years [5]. Similarly, in another study in northern Tanzania, of all patients who underwent a fiberoptic upper GI endoscopy, 18.7% were found to have evidence of UGIB [6]. Oesophageal varices and peptic ulcer disease (PUD) being the most common causes in both these studies. Studies in Tanzania have shown a mortality rate ranging between 10 and 17% of patients admitted with UGIB [5–7].

This study was done at Muhimbili National Hospital (MNH) in Tanzania, which is the largest tertiary referral hospital currently in the country. No such studies had previously been done from this setting. Only one study was found that was prospectively done to find the risk factors of mortality among patients with UGIB. This study was aimed at identifying the causes of UGIB and risk factors for mortality in these patients, which would help better risk-stratify patients and guide management more efficiently, particularly in resource limited areas.

## Methods

This was a prospective, cohort study where participants were recruited consecutively on admission from June 2015 to January 2016 and followed up for 60 days from admission.

The study population were all patients admitted to MNH due to UGIB. MNH is one of the four tertiary hospitals in the country. It also serves as a tertiary level referral for three municipal hospitals in Dar es Salaam. MNH admits patients with upper gastrointestinal bleeding primarily at Mwaisela Ward, which serves as the Internal Medicine ward for the hospital. The hospital receives approximately 25 to 30 patients a month with acute upper gastrointestinal bleeding. The patients are initially seen at the Emergency Medicine Department of the hospital and are then shifted to the medical ward after stabilization, where they are then managed accordingly. Facility for upper gastrointestinal endoscopy is available during official work hours and is performed by trained endoscopists in the department of Gastroenterology.

A target sample size of 100 patients was established based on sample size calculation with a confidence interval of 95%, a 5% margin of error and expected incidence of 10% [7].

### Data collection process

All patients were entered into the study after an informed consent. Relevant data on demographics, medical history and comorbidities was collected on admission by interviewing the study participants or their guardians if the study participants were not able to answer. Admission vital signs from the Emergency Medicine Department (blood pressure, pulse rate, respiratory rate and oxygen saturation) were also recorded. Data on comorbidities was either confirmed by the patients themselves or through their records. Blood samples for laboratory tests were collected within 24 h of admission. Upper GI endoscopy was also done by trained personnel in the Gastroenterology Unit for those who could afford to pay for it in order to identify the cause and severity of the bleeding. (Approximately $60).

All patients were then followed up while admitted in the ward, thereafter two-weekly follow-up was done after discharge up to 60 days post-initial admission or death.

UGIB was defined as any acute episode of vomiting blood or passing melena stool in the 24 h prior to admission to the hospital.

Rebleeding was defined as a separate episode of vomiting of fresh blood or melena, or nasogastric evidence of new bleeding after admission within the hospital or within 60 days post-admission.

Encephalopathy was defined as having symptoms and signs consistent with central nervous system involvement.

Chronic liver disease was defined by clinical criteria of stigmata of chronic liver disease with evidence of small liver span by clinical examination or ultrasound.

Renal insufficiency was defined as having acute kidney injury and/or chronic kidney disease from any cause.

All patients were treated according to standard treatment protocols of the hospital.

Data was analyzed using SPSS 23 for analysis. Relevant frequencies and tables were generated for all variables. Proportions and medians/interquartile ranges were calculated for appropriate variables. The differences in median values of continuous variables between the outcome groups was determined statistically by use of Mann-Whitney U test. Incidence of mortality, rebleeding and readmission were calculated. All risk factors were analyzed to determine their association with mortality by use of the chi-square test. Relative Risk was used as a measure of association for factors associated with 60-day mortality. Cox-regression analysis was done to find out the independent risk of each categorical variable towards 60-day mortality. All factors with a *p* value of <0.05 in the bivariate analysis were included in the regression model. Missing indicator variable method was used to retain cases with missing data in the regression model. Statistical significance was set at *p* value <0.05.

## Results

### Etiology of upper GI bleeding

**Fig. 1:**
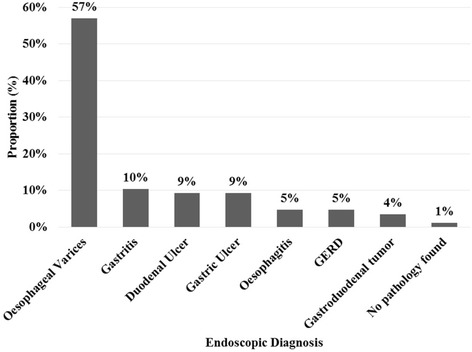
Endoscopic etiologies of UGIB among 86 patients who underwent endoscopy

From the 170 study participants, 86 underwent endoscopy. The most common cause of UGIB in these patients was oesophageal varices (57.0%), followed by peptic ulcer disease (18.6%) and gastritis (10.4%) (See Fig. 1).

Patients who did not undergo endoscopy were more likely to have renal insufficiency (OR 3.30, 95% CI 1.31–8.33, p 0.009) and encephalopathy (OR 4.17, 95% CI 1.12–15.53, p 0.023) on admission. None of the patients with HIV infection underwent endoscopy (p 0.006).

### Outcomes

Over a period of 60 days, 57 patients died (33.5%). Almost a quarter (24.6%) died in the first 24 h after admission, and almost half (49.1%) died within 72 h of admissions. Majority (96.5%) died in the first 30 days post-admission.

Rebleeding was present in 12 patients (7.1%). A quarter (25.0%) rebled within the first 96 h of admission.

Four patients were readmitted due to UGIB in the follow up period. All of them were readmitted after 30 days and before 60 days post-admission.

**Fig. 2:**
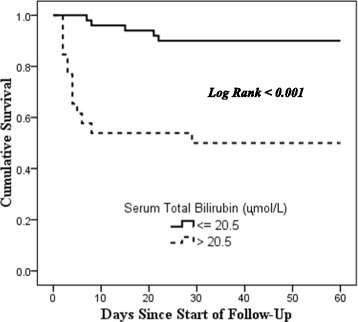
Kaplan-Meier Survival Curve for 60-day mortality by Serum Total Bilirubin Levels

**Fig. 3:**
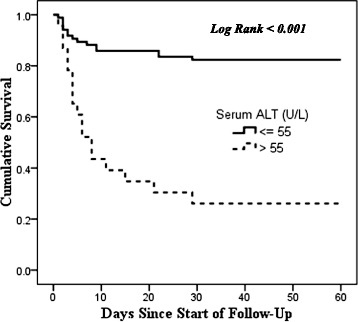
Kaplan-Meier Survival Curve for 60-day mortality by Serum ALT Levels

**Fig. 4:**
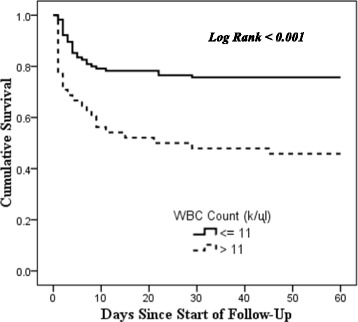
Kaplan-Meier Survival Curve for 60-day mortality by WBC Count

**Fig. 5:**
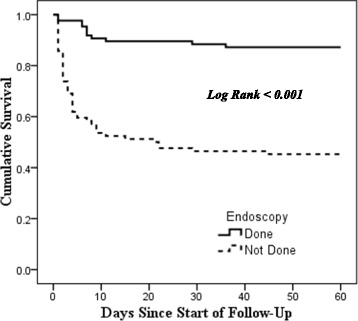
Kaplan-Meier Survival Curve for 60-day mortality by Endoscopy Status

Figures 2, 3, 4 and 5 show the Kaplan-Meier survival curves for the independent predictors of 60-day mortality. (See Figs. 2, 3, 4 and 5).

Patient population is described in Table 1. The association of mortality to different continuous variables (Table 2) and categorical variables (Table 3) revealed multiple significant associations. Factors that were significantly associated with mortality in univariate analysis (p < 0.05) were entered into the regression model for multivariate analysis as shown in Table 4.

**Table 1:** Clinical and Demographic Characteristics of Study Population

Characteristic	Median (25–75) / Frequency
Age	40.0 (31.0–56.3)
Sex	
Male	121 (71.2%)
Female	49 (28.8%)
History of previous UGIB	65 (38.2%)
Use of NSAIDs in previous one week	7 (4.1%)
Comorbidity	
Any Comorbidity	106 (62.4%)
Cardiac Disease	14 (8.2%)
Renal Insufficiency	26 (15.3%)
Chronic Liver Disease	52 (30.6%)
Encephalopathy	14 (8.2%)
Malignancy	16 (9.4%)
Hypertension	13 (7.6%)
Diabetes Mellitus	5 (2.9%)
HIV	7 (4.1%)
Smoking	9 (5.3%)
Alcohol use in previous one week	22 (12.9%)
Initial Vital Signs	
Systolic Blood Pressure (mmHg)	113.5 (102.0–130.0)
Diastolic Blood Pressure (mmHg)	63.0 (56.8–80.3)
Pulse Rate (beats/min)	101.5 (86.0–108.3)
Respiratory Rate (breaths/min)	20.0 (19.0–23.0)
Arterial Oxygen Saturation (%)	100.0 (100.0–100.0)
Initial Laboratory Data	
White Blood Count (k/μl)	8.1 (4.6–12.4)
Hemoglobin (g/dL)	6.2 (4.5–8.8)
Platelets (k/μl)	178.5 (90.8–293.3)
Serum Creatinine (μmol/L)	78.0 (63.5–150.5)
Serum BUN (g/dL)	6.5 (3.3–12.6)
Serum ALT (U/L)	23.0 (15.0–47.0)
Serum AST (U/L)	36.0 (22.0–93.0)
Serum Total Bilirubin (μmol/L)	12.7 (7.6–29.9)
Prothrombin Time (seconds)	12.5 (11.2–14.1)
APTT (seconds)	28.4 (25.2–32.8)
INR	1.1 (1.0–1.3)
Rockall Score	
Pre-Endoscopy	3.0 (1.0–4.0)
Post-Endoscopy	6.0 (4.0–7.0)

A total 170 individuals were recruited in the study. The mean and median age of the study population was 44.9 and 40.0 years, respectively. Majority were males (71.2%) and 38.2% had a previous episode of UGIB (either hematemesis or melena). Chronic liver disease was the most common comorbidity present (30.6%). (See Table 1)

**Table 2:** Demographic and Clinical Predictors for 60-day Mortality (Continuous Variables)

Factor	60-day Mortality		*p* value
	Yes (Median, 25–75)	No (Median, 25–75)	
Age	45.0 (33.5–57.5)	39.0 (31.0–55.5)	0.168
Initial Vital Signs			
Systolic Blood Pressure (mmHg)	114.0 (99.5–131.0)	116.0 (103.0–130.0)	0.513
Diastolic Blood Pressure (mmHg)	70.0 (57.5–82.5)	67.0 (56.0–79.0)	0.419
Pulse Rate (beats/min)	101.0 (91.0–113.0)	97.0 (83.5–108.0)	0.066
Respiratory Rate (breaths/min)	20.0 (19.0–23.0)	20.0 (19.5–23.0)	0.972
Arterial Oxygen Saturation (%)	100.0 (100.0–100.0)	100.0 (100.0–100.0)	*0.035*
Initial Laboratory Data			
White Blood Count (k/μl)	10.9 (7.7–16.7)	6.7 (4.0–10.2)	*<0.001*
Hemoglobin (g/dL)	7.4 (4.9–9.3)	6.0 (4.4–8.2)	0.058
Platelets (k/μl)	183.0 (133.0–278.0)	172.5 (77.4–300.3)	0.339
Serum Creatinine (μmol/L)	105.5 (72.3–289.2)	69.4 (62.2–101.6)	*0.001*
Serum BUN (g/dL)	9.7 (4.7–23.4)	4.5 (3.0–9.1)	*0.001*
Serum ALT (U/L)	58.0 (14.3–113.0)	21.0 (15.0–35.0)	*0.004*
Serum AST (U/L)	74.0 (33.5–274.0)	29.5 (18.0–50.8)	*<0.001*
Serum Total Bilirubin (μmol/L)	83.5 (17.7–421.1)	11.3 (7.0–17.6)	*0.001*
Prothrombin Time (seconds)	14.1 (12.7–19.4)	12.1 (11.0–13.3)	*0.002*
APTT (seconds)	28.5 (24.9–37.5)	28.4 (25.2–31.4)	0.533
INR	1.3 (1.2–1.7)	1.1 (1.0–1.2)	*0.001*
Rockall Score			
Pre-Endoscopy Rockall Score	3.0 (2.0–4.0)	3.0 (1.0–4.0)	0.143
Post-Endoscopy Rockall Score	6.0 (5.0–7.0)	6.0 (4.0–7.0)	0.620

Comparison of medians of different continuous variables revealed that the following were all associated with a higher risk of mortality: A raised WBC, serum creatinine, serum BUN, serum ALT, serum AST, serum total bilirubin, prothrombin time and INR and reduced oxygen saturation on admission (*p* < 0.05). (See Table 2)

**Table 3:** Demographic and Clinical Predictors for 60-day Mortality (Categorical Variables)

Factor	60-day Mortality			Relative Risk (95% CI)	*P* value
	Yes	No	Total		
Age					
> 40 years	31 (37.3%)	52 (62.7%)	83	1.25	0.303
≤ 40 years	26 (29.9%)	61 (70.1%)	87	(0.82–1.91)	
Sex					
Male	41 (33.9%)	80 (66.1%)	121	1.04	0.878
Female	16 (32.7%)	33 (67.3%)	49	(0.65–1.67)	
History of previous UGIB					
Yes	21 (27.6%)	55 (72.4%)	76	0.72	0.143
No	36 (38.3%)	58 (61.7%)	94	(0.46–1.13)	
Use of NSAIDs in previous one week					
Yes	1 (14.3%)	6 (85.7%)	7	0.42	0.426
No	56 (34.4%)	107 (65.6%)	163	(0.07–2.58)	
Any Comorbidity					
Yes	40 (37.7%)	66 (62.3%)	106	1.42	0.135
No	17 (26.6%)	47 (73.4%)	64	(0.88–2.29)	
Cardiac Disease					
Yes	6 (42.9%)	8 (57.1%)	14	1.31	0.556
No	51 (32.7%)	105 (67.3%)	156	(0.69–2.50)	
Renal Insufficiency					
Yes	16 (61.5%)	10 (38.5%)	26	*2.16*	*0.001*
No	41 (28.5%)	103 (71.5%)	144	*(1.45–3.22)*	
Chronic Liver Disease					
Yes	21 (28.0%)	54 (72.0%)	75	0.74	0.175
No	36 (37.9%)	59 (62.1%)	95	(0.47–1.15)	
Encephalopathy					
Yes	11 (78.6%)	3 (21.4%)	14	*2.67*	*<0.001*
No	46 (29.5%)	110 (70.5%)	156	*(1.85–3.84)*	
Malignancy					
Yes	9 (56.3%)	7 (43.8%)	16	*1.81*	*0.043*
No	48 (31.2%)	106 (68.8%)	154	*(1.1–2.95)*	
Hypertension					
Yes	6 (46.2%)	7 (53.8%)	13	1.42	0.364
No	51 (32.5%)	106 (67.5%)	157	(0.76–2.67)	
Diabetes Mellitus					
Yes	4 (80.0%)	1 (20.0%)	5	*2.49*	*0.044*
No	53 (32.1%)	112 (67.9%)	165	*(1.52–4.07)*	
HIV					
Yes	5 (71.4%)	2 (28.6%)	7	*2.24*	*0.043*
No	52 (31.9%)	111 (68.1%)	163	*(1.33–3.76)*	
Smoking					
Yes	3 (33.3%)	6 (66.7%)	9	0.99	1.000
No	54 (33.5%)	107 (66.5%)	161	(0.39–2.57)	
Alcohol use in previous one week					
Yes	8 (36.4%)	14 (63.6%)	22	1.10	0.763
No	49 (33.1%)	99 (66.9%)	148	(0.60–2.00)	
Systolic Blood Pressure					
< 110 mmHg	23 (37.1%)	39 (62.9%)	62	1.18	0.455
≥ 110 mmHg	34 (31.5%)	74 (68.5%)	108	(0.77–1.81)	
Diastolic Blood Pressure					
< 60 mmHg	17 (31.5%)	37 (68.5%)	54	0.91	0.700
≥ 60 mmHg	40 (34.5%)	76 (65.5%)	116	(0.57–1.46)	
Pulse Rate					
> 100 bpm	30 (38.5%)	48 (61.5%)	78	1.31	0.210
≤ 100 bpm	27 (29.3%)	65 (70.7%)	92	(0.86–2.00)	
Respiratory Rate					
> 18 bpm	44 (32.6%)	91 (67.4%)	135	0.88	0.611
≤ 18 bpm	13 (37.1%)	22 (62.9%)	35	(0.54–1.44)	
Arterial Oxygen Saturation					
< 92%	2 (50.0%)	2 (50.0%)	4	1.51	0.603
≥ 92%	55 (33.1%)	111 (66.9%)	166	(0.55–4.12)	
White Blood Count (k/μl)					
> 11.00	26 (54.2%)	22 (45.8%)	48	*2.23*	*<0.001*
≤ 11.00	28 (24.3%)	87 (75.7%)	115	*(1.47–3.37)*	
Hemoglobin (g/dL)					
< 10.00	45 (31.7%)	97 (68.3%)	142	0.74	0.310
≥ 10.00	9 (42.9%)	12 (57.1%)	21	(0.43–1.28)	
Platelets (k/μl)					
< 140	18 (26.5%)	50 (73.5%)	68	0.69	0.115
≥ 140	36 (38.3%)	58 (61.7%)	94	(0.43–1.11)	
Serum Creatinine (μmol/L)					
> 115	21 (53.8%)	18 (46.2%)	39	*2.20*	*0.001*
≤ 115	22 (24.4%)	68 (75.6%)	90	*(1.38–3.51)*	
Serum BUN (g/dL)					
> 7.4	21 (47.7%)	23 (52.3%)	44	*2.39*	*0.003*
≤ 7.4	12 (20.0%)	48 (80.0%)	60	*(1.32–4.32)*	
Serum ALT (U/L)					
> 55	17 (73.9%)	6 (26.1%)	23	*4.19*	*<0.001*
≤ 55	15 (17.6%)	70 (82.4%)	85	*(2.49–7.04)*	
Serum AST (U/L)					
> 34	24 (40.7%)	35 (59.3%)	59	*2.35*	*0.007*
≤ 34	9 (17.3%)	43 (82.7%)	52	*(1.20–4.59)*	
Serum Total Bilirubin (μmol/L)					
> 20.5	13 (50.0%)	13 (50.0%)	26	*5.00*	*<0.001*
≤ 20.5	5 (10.0%)	45 (90.0%)	50	*(2.0–12.50)*	
Prothrombin Time (seconds)					
> 12.1	13 (46.4%)	15 (53.6%)	28	*3.71*	*0.008*
≤ 12.1	3 (12.5%)	21 (87.5%)	24	*(1.20–11.51)*	
APTT (seconds)					
> 30.4	7 (35.0%)	13 (65.0%)	20	1.08	0.842
≤ 30.4	11 (32.4%)	23 (67.6%)	34	(0.50–2.34)	
INR					
> 1.13	13 (48.1%)	14 (51.9%)	27	*4.17*	*0.004*
≤ 1.13	3 (11.5%)	23 (88.5%)	26	*(1.34–12.97)*	
Endoscopy					
Not Done	46 (54.8%)	38 (45.2%)	84	*4.28*	*<0.001*
Done	11 (12.8%)	75 (87.2%)	86	*(2.39–7.69)*	
Endoscopy Diagnosis					
Oesophageal Varices	6 (12.2%)	43 (87.8%)	49	0.91	1.000
Other Diagnosis	5 (13.5%)	32 (86.5%)	37	(0.30–2.74)	
Pre-Endoscopy Rockall Score				1.29	
> 3	21 (39.6%)	32 (60.4%)	53	(0.84–1.98)	0.257
≤ 3	36 (30.8%)	81 (69.2%)	117		
Post-Endoscopy Rockall Score					
> 5	8 (15.1%)	45 (84.9%)	53	1.61	0.524
≤ 5	3 (9.4%)	29 (90.6%)	32	(0.46–5.63)	

Mortality was also compared to different categorical variables as outlined in Table 3. A high serum total bilirubin was associated with a 5-fold increased risk of mortality (RR = 5.00, *p* < 0.001). Patients with a raised serum ALT level (RR = 4.19, *p* < 0.001), a raised INR (RR = 4.17, *p* = 0.004), a prolonged prothrombin time (RR = 3.71, *p* = 0.008) and those who did not undergo endoscopy during admission (RR = 4.28, *p* < 0.001) had an almost 4-fold increased risk of mortality within 60 days from admission. Other factors that were associated with a higher risk of mortality included patients with renal insufficiency (RR = 2.16, *p* < 0.001), encephalopathy (RR = 2.67, *p* < 0.001), malignancy (RR = 1.81, *p* = 0.043), diabetes mellitus (RR = 2.49, *p* = 0.044), HIV disease (RR = 2.24, *p* = 0.043), a raised WBC count (RR = 2.23, *p* < 0.001), a raised serum creatinine level (RR = 2.20, *p* = 0.001), a raised serum BUN level (RR = 2.39, *p* = 0.003), and a raised serum AST level (RR = 2.35, *p* = 0.007). (See Table 3)

**Table 4:** Cox-Regression Analysis of Predictors for 60-day Mortality (Multivariate Analysis)

Predictor	Hazard Ratio (95% CI)	*P* value
Renal Insufficiency	1.38 (0.54–3.51)	0.504
Encephalopathy	1.19 (0.46–3.06)	0.723
HIV	0.58 (0.16–2.07)	0.402
Diabetes Mellitus	2.14 (0.54–8.50)	0.279
Malignancy	0.58 (0.21–1.64)	0.303
White Blood Cell Count >11 k/μl	*2.45 (1.23–4.89)*	*0.011*
Serum Creatinine >115 μmol/L	1.55 (0.57–4.23)	0.896
Serum BUN >7.4 g/dL	1.46 (0.55–3.85)	0.445
Serum ALT >55 U/L	*4.22 (1.31–13.57)*	*0.016*
Serum AST > 34 U/L	1.11 (0.39–3.17)	0.843
Serum Total Bilirubin >20.5 μmol/L	*5.79 (1.58–21.25)*	*0.008*
Prothrombin Time > 12.1 s	0.33 (0.01–21.14)	0.598
INR > 1.13	8.57 (0.14–536.04)	0.309
Endoscopy Not Done	*4.40 (2.11–9.17)*	*<0.001*

Multivariate analysis to identify independent predictors of 60-day mortality was done using Cox-Regression analysis. Factors that were shown to be significantly associated with a higher risk of 60-day mortality by bivariate analysis were included in the regression model. Multivariate analysis showed that a higher WBC count of >11 k/μl, a high serum ALT >55 U/L, a high serum total bilirubin >20.5 μmol/L were independently associated with an increased risk of mortality. Patients who did not undergo endoscopy had a 4.4 times higher rate of death within 60 days of admission (Table 4)

## Discussion

This prospective, cohort study aimed at identifying the major causes of upper gastrointestinal bleeding and the magnitude of mortality, rebleeding and readmission and their risk factors in a tertiary level hospital in Tanzania.

The most common cause of upper GI bleeding in this study group was oesophageal varices, found in 57% of patients. PUD accounted for 18% of cases (equally distributed among duodenal and gastric ulcer). The high prevalence of oesophageal varices has also been found in studies done in other regions of Tanzania such as Moshi [5] and Mwanza [6, 7]. In contrast, PUD was the most common cause of UGIB in studies done in Korea [8], USA [9] and various European countries [3, 10].

Oesophageal varices are most often a result of portal hypertension. Schistosomiasis and chronic liver disease are among the most common causes of portal hypertension in sub-Saharan Africa. The prevalence of chronic liver disease is similar to studies done in Romania [3, 4]. However, this prevalence is higher than those found in studies in Mwanza [6, 7], Europe [11] and Korea [8, 12]. One reason for this could be the type of patients involved in this study. The study was conducted in a tertiary referral center, hence it admits patients with the most severe forms of disease. Secondly, although not directly tested, the high prevalence of schistosomiasis and chronic hepatitis B infection in this setting may have contributed to the high prevalence of chronic liver disease. More than 90% of cases of schistosomiasis occur in sub-Saharan Africa and Tanzania is the second highest in terms of burden of disease in this region [13]. The estimated country prevalence of schistosomiasis is 51.5% [13]. One study done in Mwanza showed a high rate of active schistosome infection in adults presenting with hematemesis [7]. Similarly, one study found a 12.4% positivity of *S. mansoni* in stool samples [14] Studies in Tanzania have shown a prevalence of chronic hepatitis B infection ranging from 2.9–7.0% [15–17].

PUD was the second most common diagnosis. It is well known that PUD is associated with *H. pylori* infection*.* Studies done in Tanzania have shown a prevalence of *H. pylori* to be more than 85% in the population [18–20]. The prevalence was shown to increase with age [19].

The overall mortality rate within 60 days of admission was 33.5%. This is very high compared to the overall mortality rate from upper GI bleeding worldwide [2]. Many studies looking at mortality rates among patients with upper GI bleeding have shown mortality rates ranging from 1 to 20% [3, 6, 7, 9, 21–24]. A few studies have mortality rates higher than 30% [10, 25, 26].

The high mortality rate in this study could be due to delayed or more severe presentation to a referral center, thus increasing the likelihood of mortality due to delay or failure in achieving hemodynamic stability, particularly in the absence of urgent endoscopic intervention. Furthermore, there is a general lack of available intervention measures such as blood transfusion and urgent endoscopic intervention and this may have contributed to the high mortality. It is noteworthy that almost half of the patients died within 72 h of admission to the hospital, suggesting the importance of early intervention as the key in the management of patients with acute UGIB.

A high WBC count was associated with almost 2.5 times higher rate of death compared to patients with a normal WBC count on admission. A high WBC has been associated with mortality in other studies too [27–29]. Leukocytosis is a marker for inflammation and infection, but it also occurs in other situations like trauma, exercise, drug therapy with steroids, malignancy, poisoning, psychosis and diabetic ketoacidosis. Circulating catecholamines may also lead to leukocytosis. It has been speculated that leukocytosis may represent an acute phase marker [30]. One study on upper GI bleeding patients revealed that a high WBC count was associated with more severe presentation and disease course, although it was not associated with a higher risk of mortality. The authors used a lower cut-off value of high WBC (8.5 k/μl) [31] Several studies have shown the association between leukocytosis and higher risk of mortality in various conditions [32–34]. It is possible that some underlying infection or inflammatory condition contributed to the mortality in these patients.

Patients admitted with elevated ALT had about 4 times higher rate of death within 60 days compared to patients admitted with normal levels of ALT. Abnormal liver enzymes, particularly a raised ALT has been shown to be a risk factor for mortality in different studies [3, 24, 35, 36].

ALT levels can be raised in patients with myocardial infarction and with ischemia to the liver [37]. Although these were not measured directly, it is possible that more severe bleeding was associated with ischemia to the heart and liver, thus contributing to the higher mortality. Higher levels of ALT have also been associated with obesity, serum cholesterol and underlying unrecognized liver diseases [37, 38]. It may be possible that these comorbidities may have contributed to mortality in this group of patients.

A high serum total bilirubin during admission was associated with an almost 6 times increased rate of death in this study population. Other studies have also shown this association between high serum total bilirubin and mortality [21, 22, 25, 31, 36, 39, 40]. High levels of serum total bilirubin have been associated with all-cause mortality [41, 42]. The exact mechanism is unclear. It has been shown in different studies that a high level of serum total bilirubin is associated with cardiovascular disease, particularly ischemic heart disease [43] and acute myocardial infarction [44]. The increased risk of acute cardiovascular events in patients with high serum total bilirubin may have contributed to the increased mortality rate in this study group.

Patients who did not undergo endoscopy during their hospital stay had about 4 times a higher rate of death within 60 days compared to patients who had an endoscopy done. Lack of endoscopy was shown to be a risk factor for mortality in another study done in Bugando [7]. It may be possible that patients who were hemodynamically unstable and very sick overall on admission did not undergo endoscopy and were at higher risk of mortality, as seen by the fact that patients with renal insufficiency, HIV and encephalopathy were all less likely to undergo endoscopy. Consensus guidelines outline the importance of endoscopy in acute upper GI bleeding. It helps to identify the source of bleeding, provide prognostic information regarding the risk of bleeding, and offer therapy for hemostasis [45]. One study also showed reduced incidence of myocardial infarction and overall mortality in patients who received initial resuscitation (including endoscopic intervention) [46] Hence, the patients who did not undergo endoscopy may have missed the chance to be offered endoscopic intervention because of a lack of diagnosis and this may be the reason for the increased mortality rate in this group of patients.

The strength of this study was that it was a prospective study where many variables that could potentially predict adverse outcomes were assessed. Furthermore, all patients were followed-up in this study.

The study had some limitations. Half of the patients did not undergo endoscopy due to not being able to afford it. This may have underestimated the burden of some etiologies. Importantly, not undergoing endoscopy was an independent predictor of mortality. Therefore, more serious etiologies may have been missed which led to early death. Some laboratory values were not obtained for some patients and this may have underestimated or overestimated the significance of these factors with the outcomes. Presence of comorbidities was assessed by patient interview or clinical diagnosis. True melena was ascertained verbally only. This may have included some patients who had black stool from ingestion of previously prescribed ferrous sulphate. Gold standard criteria for diagnosis of comorbidities were not used in all patients. This study identified an important area in the local country but the results may not necessarily be generalisable to settings with different epidemiological disease patterns, particularly for chronic liver disease. Subgroup analysis to identify specific risk factors for mortality following variceal bleeding and ulcer bleeding could not be done due to small sample size these subgroups would have.

## Conclusions

Oesophageal varices was the most common cause of UGIB, followed by peptic ulcer disease. There was a high burden of mortality in this study, about one-third of patients admitted with UGIB died within 60 days. Rebleeding and readmission rates were low. Independent predictors of 60-day mortality were a high WBC count, raised serum ALT, raised serum total bilirubin and a lack of endoscopy. It is recommended that patients require early and more aggressive intervention in acute UGIB and possibly larger studies can be conducted looking at other factors that may contribute to mortality in UGIB patients. It is also important to study the reasons for a lack of endoscopy among patients with UGIB since this is a potentially correctable factor which can then lead to reduction in mortality and improvement on clinical care of these patients in the country.

## Abbreviations

ALT: Alanine Aminotransferase
aPTT: Activated Partial Thromboplastin Time
AST: Aspartate Aminotransferase
BUN: Blood Urea Nitrogen
HIV: Human Immunodeficiency Virus
HR: Heart Rate
INR: International Normalized Ratio
MNH: Muhimbili National Hospital
MUHAS: Muhimbili University of Health and Allied Sciences
PT: Prothrombin Time
SBP: Systolic Blood Pressure
UGIB: Upper Gastrointestinal Bleeding
